# KAT6A–inhibitor co-crystal structures: tackling a challenging crystallization target via two alternative approaches

**DOI:** 10.1107/S2059798326008545

**Published:** 2026-08-27

**Authors:** Vera Puetter, Léa Bouché, Katrin Nowak-Reppel, Steven J. Ferrara, Stefan N. Gradl, Daniel Korr, Craig A. Strathdee, Antonius ter Laak, Roman C. Hillig

**Affiliations:** a Nuvisan ICB GmbH, Muellerstrasse 178, 13353Berlin, Germany; b Research & Development, Pharmaceuticals, Bayer AG, Müllerstrasse 178, 13353Berlin, Germany; c Broad Institute, Center for the Development of Therapeutics, Broad Institute of MIT and Harvard, Cambridge, Massachusetts, USA; F. Hoffmann-La Roche Ltd, Switzerland

**Keywords:** surface modification, surrogate approach, back-soaking, small-molecule inhibitors, structure-based drug discovery, back soaking

## Abstract

The histone lysine acetyltransferase KAT6A belongs to the MYST family of lysine acetyltransferases which consists of five isoforms. KAT6A (and its paralog KAT6B) have emerged as a promising epigenetic drug targets in cancer, supported by recent clinical data. As support for our high-throughput screening approach aimed at discovering new inhibitors of KAT6A, we developed a crystallization platform system termed KAT6A^mutCys^ in which four surface cysteine residues of KAT6A were mutated to serine. This construct crystallizes readily when in complex with its cofactor AcCoA. The obtained co-crystals were successfully used in a back-soaking approach in which AcCoA was soaked out of the crystals and small-molecule inhibitors were soaked in. In parallel, we explored a previously published surrogate approach termed MYST^cryst^ in which active-site residues of the related, but easier to crystallize, enzyme KAT8 were mutated to those found in KAT6A. By comparing co-crystal structures of the same ligand bound to KAT6A from our Cys-to-Ser approach and to mutated KAT8 (MYST^cryst^) we found that the KAT8 surrogate approach indeed successfully reproduced the binding mode observed in the KAT6A structure, while delivering structures with significantly higher resolution. The MYST^cryst^ approach was therefore employed to determine the binding modes of two further small-molecule inhibitors, both from our KAT6A inhibitor optimization program and from a competitor lead series. These structures reveal alternative inhibitor conformations at the binding-site entry and the importance of filling a hydrophobic subpocket in the interior of the binding pocket. These insights will aid future efforts towards the development of KAT6A inhibitors as anticancer drugs.

## Abstract 2

We report a novel cysteine-to-serine surface-mutation approach to determine co-crystal structures of KAT6A with small-molecule inhibitors and compare it with the previously published MYST^cryst^ approach which uses mutated KAT8 as a surrogate for KAT6A. A series of co-crystal structures obtained from both approaches, with three different inhibitor chemotypes, reveal alternative inhibitor conformations at the binding-site entry and the importance of filling a hydrophobic subpocket. These insights expand the experimental basis for future KAT6A inhibitor design for cancer therapy.

## 1. Introduction

Lysine acetyltransferases (KATs) catalyse the acetylation of the side chains of lysine residues using the cofactor acetyl-coenzyme A (AcCoA). They thus act as epigenetic writers and as important regulators in chromatin organization and function (Arrowsmith *et al.*, 2012 ▸). In recent years, KATs have been identified as an important class of potential therapeutic targets, especially in the field of cancer (Huang *et al.*, 2019 ▸; Sheikh & Akhtar, 2019 ▸; White *et al.*, 2024 ▸). Within the lysine acetyltransferase family, KAT6A belongs to the MYST subfamily, which was named after its founding members MOZ, YBF2/SAS3, SAS2 and TIP60 and comprises the proteins KAT5, KAT6A (MYST3), KAT6B (MYST4), KAT7 (MYST2) and KAT8 (MYST1) (Hirsch *et al.*, 2017 ▸; Voss & Thomas, 2018 ▸). Within this subfamily, KAT6A and KAT6B are two closely related isoforms which both form a complex with the bromodomain–PHD finger protein (BRPF1/2/3), inhibitor of growth 5 (ING5) and MYST/Esa-associated factor 6 (MEAF6) and both modulate downstream gene expression by acetylating histone 3 on lysine residue 9 (H3K9) and especially on lysine residue 23 (H3K23) (Lian *et al.*, 2020 ▸; Lv *et al.*, 2017 ▸; Yang & Ullah, 2007 ▸; Wiesel-Motiuk & Assaraf, 2020 ▸). KAT6A and KAT6B genes have been frequently found to be amplified in cancer and their function has been investigated in breast cancer, bladder urothelial carcinoma, lung adenocarcinoma, oesophageal carcinoma, colon adenocarcinoma, ovarian serous cystadenocarcinoma, uterine corpus endometrial carcinoma, KAT6A-rearranged AML and uterine carcinosarcoma (Jabs *et al.*, 2017 ▸; Berger *et al.*, 2018 ▸; Gelsi-Boyer *et al.*, 2005 ▸; Hu *et al.*, 2019 ▸; Sheridan *et al.*, 2024 ▸; Olsen *et al.*, 2025 ▸; Liang *et al.*, 2025 ▸). In breast cancer, where translational research is most advanced, KAT6A/B catalytic inhibitors are being evaluated in early clinical studies, providing evidence that disease biology depends on KAT6A/B acetyltransferase activity (Mukohara *et al.*, 2024 ▸; Roussel-Simonin *et al.*, 2025 ▸). An overview of KAT6A/B biology and recent inhibitor development can be found in three recent publications (Suwandi *et al.*, 2026 ▸; Xi *et al.*, 2026 ▸; Mukohara, 2026 ▸).

The first reported crystal structures of the catalytic domain of KAT6A, PDB entries 2rc4 (Holbert *et al.*, 2007 ▸) and 2ozu (Structural Genomics Consortium, unpublished work), showed KAT6A in complex with its cofactor AcCoA and revealed an elongated and deep cofactor-binding site located between two lobes of the catalytic domain. The substrate lysine residue inserts into a shallow funnel-shaped binding site at one end of the elongated binding site, where the lysine side-chain amino group takes over the acetyl group from a catalytic cysteine residue (Cys646) which was previously acetylated by AcCoA (Yan *et al.*, 2002 ▸). When we started this project, KAT6A as well as all other available crystal structures of MYST family members had been crystallized only in complex with AcCoA, and no co-complex crystal structures with a small-molecule inhibitor had been reported. This changed in 2018 when Baell and coworkers published the first crystal structure of a mutated form of KAT8 (termed MYST^cryst^) in complex with the KAT6A inhibitor WM-8014, a small-molecule inhibitor which is selective for the two closely related family members KAT6A and KAT6B (PDB entry 6ba2; Baell *et al.*, 2018 ▸). This was followed by KAT6A co-crystal structures with the inhibitor CTX-648/PF-9363 from Pfizer (PDB entry 8dd5; Sharma *et al.*, 2023 ▸), which is a structurally close relative of the clinical candidate PF-07248144/Prifetrastat (Mukohara *et al.*, 2024 ▸; Sommerhalder *et al.*, 2023 ▸), and by the crystal structure of KAT6A in complex with BAY-184 (PDB entry 9fkr; ter Laak *et al.*, 2024 ▸) and of mutated KAT8 (MYST^cryst^) in complex with the optimized inhibitors BAY-7728 (PDB entry 29tm) and compound 38 (PDB entry 29tn) (ter Laak *et al.*, 2026 ▸).

Here, we report the rationale behind the KAT6A surface-mutation approach we previously used for the characterization of BAY-184 (ter Laak *et al.*, 2024 ▸), together with additional co-complex structures generated that also use this new KAT6A surface-mutant approach (termed KAT6A^mutCys^ hereafter). In addition, we investigated the MYST^cryst^/KAT8^mutant^ approach developed by Stefan Hermann in the team of Michael Parker (Baell *et al.*, 2018 ▸) and report two further co-crystal structures of MYST^cryst^ in complex with KAT6A inhibitors. The inhibitor co-crystal structures presented here provide a broadened structural basis for future KAT6A inhibitor design.

## 2. Materials and methods

### 2.1. Biochemical KAT6A activity assay

The KAT6A inhibitory activity of the various compounds was quantified using the time-resolved fluorescence energy transfer (TR-FRET) assay as described in ter Laak *et al.* (2024 ▸). This assay measures the acetylation of a histone H4-derived peptide with amino-acid sequence SGRGKGGKGLGKGGAKRHRKVLRDK-biotin (Biosyntan GmbH, Berlin, GER) by recombinant human His-tagged KAT6A protein (amino acids 194–810) purified from baculovirus-infected insect cells (Sf9).

### 2.2. Protein purification, crystallization and crystal structure determination of KAT6A^mutCys^

Human KAT6A^mutCys^ (UniProt accession code Q92794, residues 509–708 with the point mutations C638S, C646S, C723S and C773S) was expressed and purified as described previously (ter Laak *et al.*, 2024 ▸). In brief, the gene was expressed in High Five insect cells with an N-terminal Flag tag, and the resulting protein was purified via affinity chromatography (Flag M2 Agarose), tag cleavage and gel filtration (Superdex S200, final buffer 25 m*M* Tris pH 8, 500 m*M* NaCl, 5% glycerol). The peak fractions were concentrated to 7.1 mg ml^−1^, supplemented with 4 m*M* AcCoA (from a 45 m*M* stock in protein buffer) and crystallized using the hanging-drop method. Drops were made from 0.8 µl protein solution mixed with 0.8 µl reservoir buffer [100 m*M* HEPES pH 7.1–7.5, 17–21%(*w*/*v*) PEG 3350, 100 m*M* sodium acetate], streak-seeded with initial crystals obtained under the same conditions before and stored at 293 K. Plate-shaped crystals grew within 3–7 weeks (Fig. 2*a*). The crystals were washed with reservoir solution, back-soaked for 9–10 days in a stepwise fashion with increasing concentrations of the respective small-molecule inhibitor (typically 1 day at 5 m*M*, 1 day at 7 m*M*, 7–8 days at 10 m*M* ligand concentration from a 100 m*M* ligand stock in protein buffer), briefly immersed in reservoir buffer supplemented with 20% glycerol and cryo-cooled in liquid nitrogen. Diffraction data sets were collected at 100 K on beamline 14.1 at the BESSY II electron-storage ring operated by the Helmholtz-Zentrum Berlin (Mueller *et al.*, 2015 ▸; Table 1 ▸). The data sets were processed using the programs *XDS* (Kabsch, 2010 ▸) and *XDSAPP* (Sparta *et al.*, 2016 ▸). Molecular replacement was carried out using *Phaser* (McCoy *et al.*, 2007 ▸) with PDB entry 2ozu as the search model. The structures were refined using *REFMAC*5 (Kovalevskiy* et al.*, 2018 ▸) and rebuilt using *Coot* (Emsley *et al.*, 2010 ▸). While the initial KAT6A^mutCys^ crystal form grown in the presence of AcCoA belonged to space group *P*1 with four KAT6A^mutCys^ protomers per asymmetric unit (chains *A*–*D*), crystals subjected to this back-soaking procedure underwent a change with a new *c* axis which was only half the length of the original *c* axis (Table 1 ▸) and with only two KAT6A protomers (chains *A* and *B*) per asymmetric unit.

### 2.3. Small-molecule inhibitor synthesis

The inhibitor WM-8014 (Baell *et al.*, 2018 ▸; Leaver *et al.*, 2019 ▸) was synthesized as described in patent application WO 2016/198507 (Voss *et al.*, 2016 ▸). The inhibitor Example 41 was synthesized as described in patent application WO 2019/043139 (Morrow *et al.*, 2018 ▸). The inhibitors compound 1 and compound 2 were synthesized as described in patent application WO 2020/216701 (Bouche *et al.*, 2019 ▸).

### 2.4. Protein purification, crystallization and crystal structure determination of KAT8^mutant^/MYST^cryst^

Human KAT8^mutant^ [UniProt Accession code Q9H7Z6, residues 174–448 with the point mutations Y95H (KAT6A numbering: His579), A161S (KAT6A numbering: Ser645), L164M (KAT6A numbering: Met648), T165I (KAT6A numbering: Ile649), K176R (KAT6A numbering: Arg660), T213S (KAT6A numbering: Ser697) and I216N (KAT6A numbering: Asn702)], with an N-terminal hexahistidine tag followed by a thrombin cleavage site, was cloned into pET-28a and expressed in *Escherichia coli*. After cell lysis, the supernatant was subjected to an IMAC purification step (5 ml HisTrap FF Crude). The corresponding fractions were pooled, concentrated to 6 mg ml^−1^ and subjected to a size-exclusion chromatography step using Superdex S200 (final buffer 50 m*M*sodium citrate pH 6.5, 200 m*M* NaCl, 5 m*M* DTT). KAT8^mutant^ eluted as a monomer and was concentrated to 10.3 mg ml^−1^ and shock-frozen in liquid nitrogen.

For co-crystal structures of KAT8^mutant^ in complex with small-molecule inhibitors, KAT8^mutant^ vials were thawed, supplemented with 1 m*M* inhibitor (from a 200 m*M* stock solution in DMSO) and incubated for 2 h at 293 K. Crystallization screens were set up at 293 K using six different commercially available initial screens and the sitting-drop method with drops made from 200 nl protein–inhibitor solution and 200 nl reservoir solution. For compound 2, the reservoir buffer was 32%(*v*/*v*) PEG 400, 0.8 *M* LiCl, 0.1 *M* Tris pH 8.5; for Example 41, the reservoir buffer was 25%(*w*/*v*) PEG 3350, 0.2 *M* NaCl, 0.1 *M* bis-Tris pH 5.5. KAT8^mutant^–ligand co-crystals from initial screens could often directly be used for crystal structure determination. Otherwise, they required one round of fine-screening to optimize the crystal size for data collection. Crystals grew usually within two to seven days as sticks or plates (Fig. 2*b*) and were cryocooled using the respective reservoir solution supplemented with 15% glycerol as a cryoprotectant. Data-collection and refinement statistics are given in Tables 1 ▸ and 2 ▸. Diffraction data sets were collected at 100 K on beamline P11 at PETRA III at the Deutsches Elektronen-Synchrotron in Hamburg, Germany (see Table 1 ▸). The data sets were processed using *XDS* (Kabsch, 2010 ▸) and *XDSAPP* (Sparta *et al.*, 2016 ▸). Molecular replacement was carried out using *Phaser* (McCoy *et al.*, 2007 ▸) with PDB entry 6ba4 (Baell *et al.*, 2018 ▸) as the search model. The structures were refined using *REFMAC*5 (Kovalevskiy* et al.*, 2018 ▸) and rebuilt using *Coot* (Emsley *et al.*, 2010 ▸). All KAT8^mutant^ data sets belonged to the same crystal form with space group *P*2_1_2_1_2_1_, with almost identical unit-cell dimensions (Table 1 ▸) and one KAT8^mutant^ protomer per asymmetric unit.

## 3. Results and discussion

### 3.1. KAT6A construct design, crystallization and back-soaking

**Figure 1:**
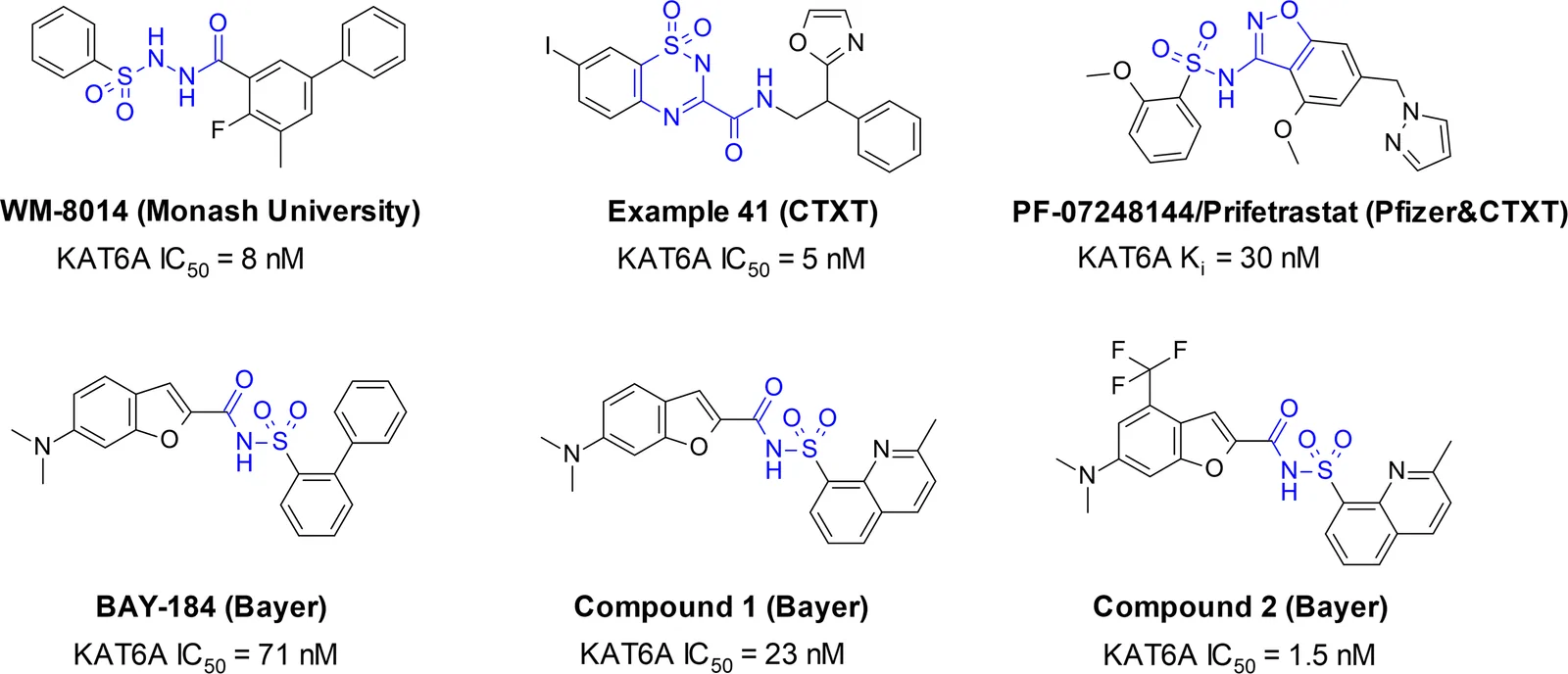
Selected examples of KAT6A/B inhibitors discussed in this study. IC_50_ values listed here are for inhibition of KAT6A, but due to the high similarity within the inhibitor-binding site, all of these inhibitors also inhibit KAT6B. IC_50_ values for WM-8014 are from Baell *et al.* (2018 ▸), that for the 1,2,4-thiadiazine Example 41 is from Morrow *et al.* (2018 ▸) (this compound is also called compound 27n in Suwandi *et al.*, 2026 ▸), the *K*_i_ value for PF-07248144/Prifetrastat is from Bozikis *et al.* (2019 ▸), the IC_50_ value for BAY-184 is from ter Laak *et al.* (2024 ▸) and those for compound 1 and compound 2 are from this study.

At the start of this work, only crystal structures of KAT6A in complex with the cofactor acetyl-CoA (AcCoA) were available (PDB entries 2rc4 and 2ozu; Holbert *et al.*, 2007 ▸; Structural Genomics Consortium, unpublished work). We first expressed the exact published expression constructs used in those studies, purified the KAT6A proteins and tested them in the presence of 1–2 m*M* AcCoA in crystallization trials, but did not obtain any crystals. Assuming that addition of a highly potent inhibitor may further stabilize an otherwise too flexible KAT6A protein, we also tested co-crystallization in the presence of the tool compound WM-8014 (Fig. 1 ▸) which had been reported at this time (Voss *et al.*, 2016 ▸; Baell *et al.*, 2018 ▸). We synthesized this compound as a tool to support our crystallization efforts and to understand the binding pocket of KAT6A. However, despite its high potency in our biochemical and biophysics assays [biochemical IC_50_ = 12 n*M*, *K*_d_(SPR) = 9.7 n*M*; ter Laak *et al.*, 2024 ▸] and a thermal stabilization of Δ*T*_m_ = 10 K in the thermal shift assay (see the supporting information to ter Laak *et al.*, 2024 ▸), the presence of WM-8014 did not trigger crystal formation either. In line with this outcome, the Structural Genomics Consortium (SGC) reported that the crystals used for the structure determination of PDB entry 2ozu were only obtained after post-translational treatment of the purified protein with iodoacetamide, which resulted in the covalent modification of four surface cysteine residues (Structural Genomics Consortium, unpublished work). (PDB entry 2ozu is a crystal structure from the SGC structural genomics project. It has not yet been published in a peer-reviewed journal. We therefore cite the PDB entry here. The protocols detailing the iodoacetamide modifications are available from the respective Structural Genomics Consortium legacy protocols website; https://public.thesgc.org/legacy_protocols/HTML/2OZU.html). Inspection of PDB entry2ozu showed that of the ten cysteine residues present in the crystallized acetylase domain construct of KAT6A, only the four cysteine residues which are solvent-accessible were modified (Cys638, Cys646, Cys723 and Cys773). Of the other six non-solvent-exposed cysteine residues, three are part of a zinc-finger motif and coordinate a structurally important zinc ion. Solvent-exposed cysteine residues can introduce heterogeneity, for example via partial oxidation or via aggregation via disulfide-bond formation. The fact that the crystals resulting in PDB entry 2ozu were only obtained when these four solvent-exposed cysteine residues were covalently modified with iodoacetamide strongly indicated that these cysteine residues may have been responsible for the failed crystallization trials. We therefore generated a new construct in which these four solvent-exposed cysteine residues were mutated to serine.

**Figure 2:**
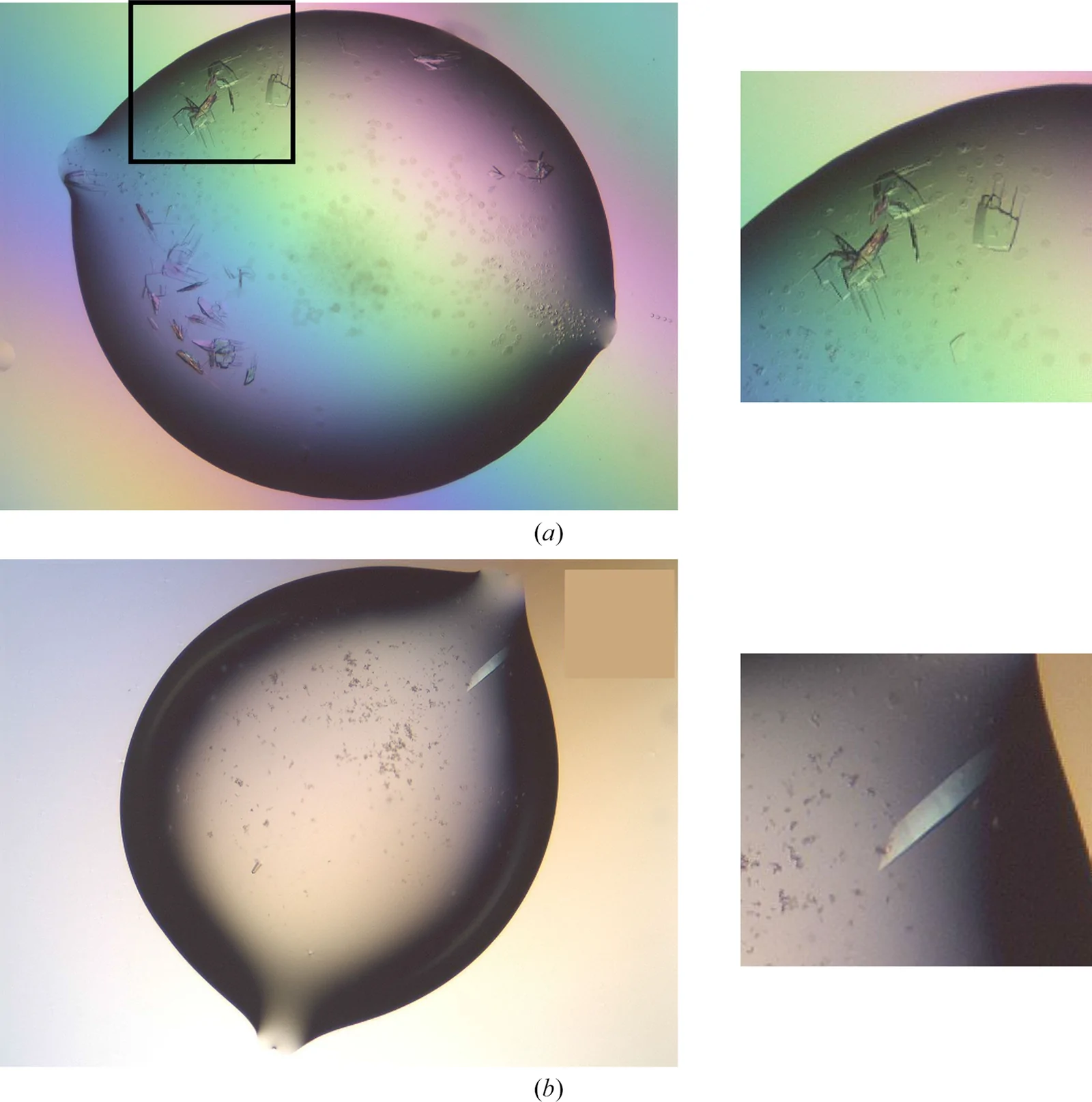
Crystals of KAT6A^mutCys^ and of KAT8^mutant^/MYST^cryst^. (*a*) shows typical plate-shaped crystals of KAT6A^mutCys^ in complex with AcCoA obtained after one round of streak-seeding. (*b*) shows a representative KAT8^mutant^/MYST^cryst^–inhibitor co-crystal obtained in a streak-seeded fine-screen, here with the lead compound BAY-7728 (ter Laak *et al.*, 2026 ▸).

**Figure 3:**
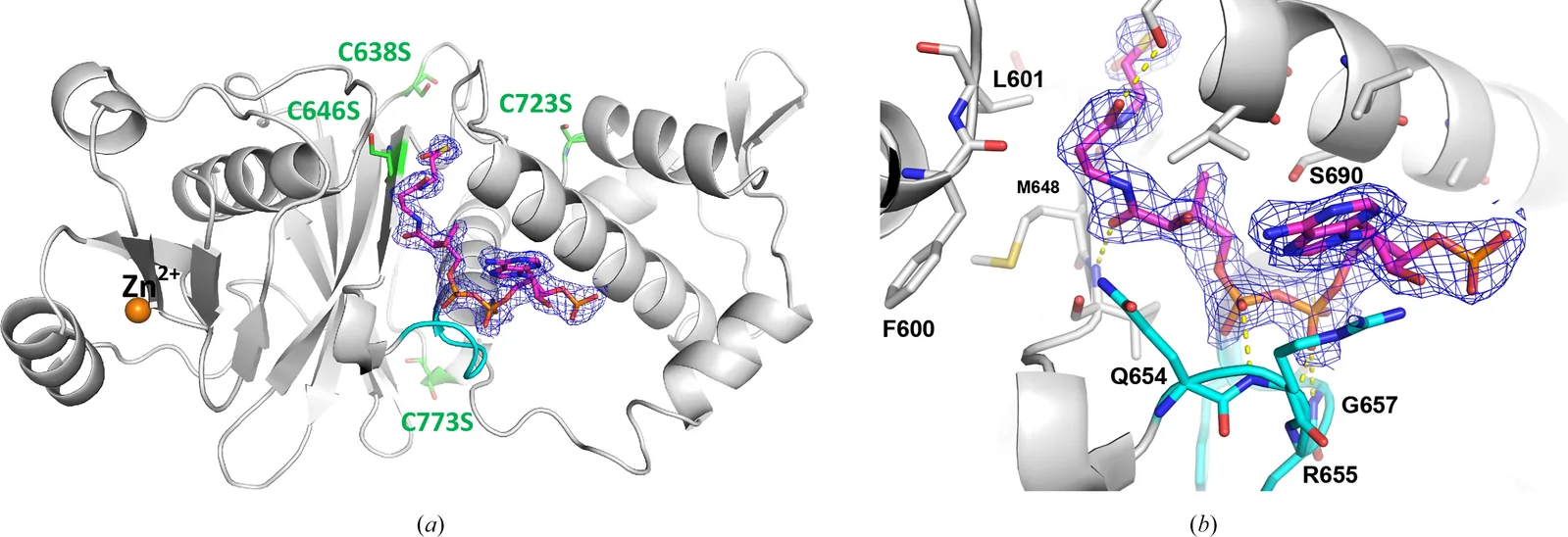
Crystal structure of KAT6A^mutCys^ in complex with AcCoA. (*a*) shows the overall fold of KAT6A^mutCys^ (PDB entry 29uq), with the four mutated surface Cys-to-Ser residues shown in stick representation with green C atoms, the co-crystallized cofactor AcCoA with magenta C atoms and the structural zinc ion as an orange sphere. Highlighted in cyan is the P-loop of KAT6A, which wraps around and coordinates the pyrophosphate unit of AcCoA via a series of hydrogen bonds. (*b*) shows a zoom into the part of the AcCoA-binding pocket where the pyrophosphate unit is coordinated within the P-loop. Residues lining this binding pocket are shown in stick representation. The 2.1 Å resolution 2*F*_o_ − *F*_c_ electron-density map around the bound cofactor (contoured at 1.3σ) is shown in blue.

This new construct KAT6A^mutCys^ expressed well in insect cells, and the purified KAT6A^mutCys^ protein finally produced crystals when pre-incubated with AcCoA (Fig. 2 ▸*a*). A data set to 2.1 Å resolution was collected and the structure (Fig. 3 ▸*a*) was solved by molecular replacement. Fig. 3 ▸(*a*) also shows the positions of the four Cys-to-Ser mutations. The structure was in principle identical to that of the wild-type KAT6A protein in complex with AcCoA (the r.m.s.d. over all C^α^ atoms of chain *A* was 0.69 Å to PDB entry 2ozu and 0.76 Å to PDB entry 2rc4). This high level of structural similarity confirms that the four Cys-to-Ser point mutations do not alter the overall fold and the cofactor-binding site, and can therefore be used as a more technically suitable surrogate for KAT6A structure-based drug design. As observed previously in PDB entry 2ozu, the side-chain amine group of Lys604 in the immediate vicinity of the catalytic cysteine residue Cys646 (mutated to Ser646 in KAT6A^mutCys^) is acetylated, suggesting that this residue may have been auto-acetylated either during insect-cell expression or incubation of the purified protein with AcCoA. The solvent-exposed adenosine group of AcCoA adopts the same conformation as in PDB entry 2ozu, which differs from that observed in PDB entry 2rc4.

**Figure 4:**
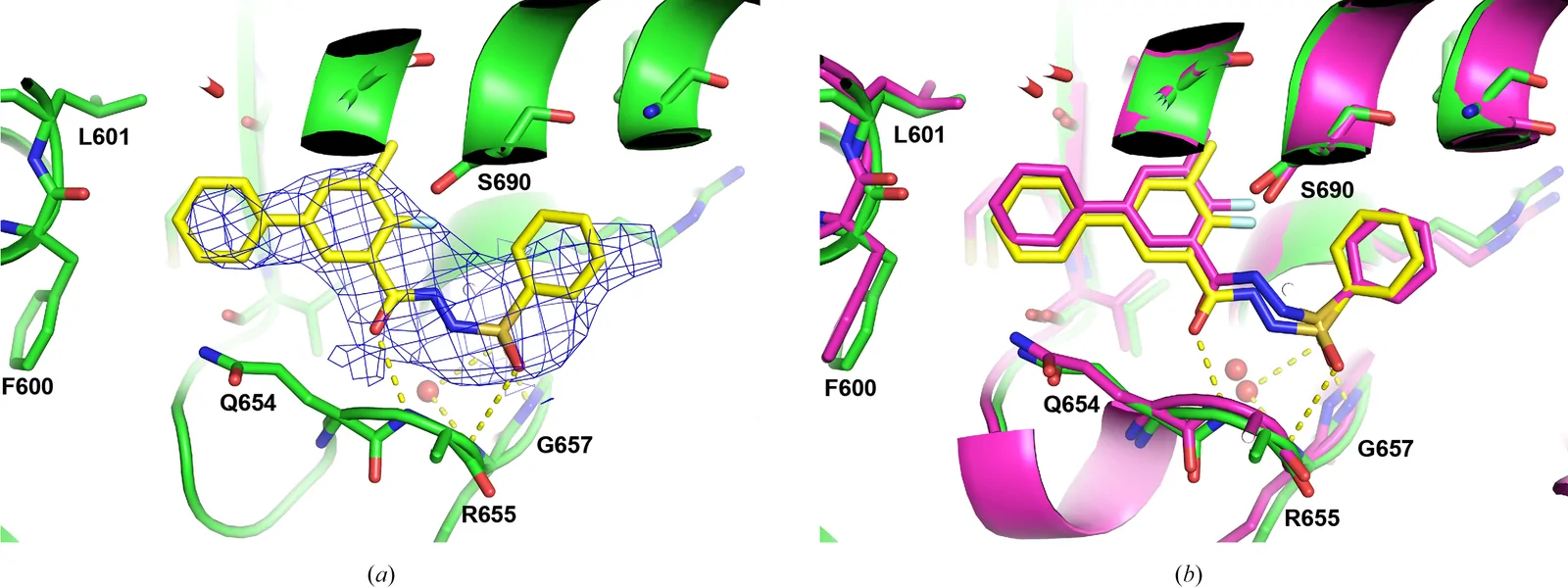
Co-crystal structure of KAT6A^mutCys^ in complex with WM-8014. (*a*) shows the tool compound WM-8014 (stick representation, C atoms in yellow) bound within the AcCoA-binding site of KAT6A (PDB entry 29ur, chain *B*). Residues lining the inhibitor-binding site are drawn in stick representation. The final 2*F*_o_ − *F*_c_ electron-density map contoured at 1.0σ is shown in blue. (*b*) shows the same view, now superimposed with the co-crystal structure of the co-complex of the same inhibitor in complex bound to KAT8^mutant^/MYST^cryst^ (PDB entry 6ba2; Baell *et al.*, 2018 ▸).

Next, we tested KAT6A^mutCys^ in co-crystallization screens with the tool compound WM-8014, but again these direct co-crystallization experiments did not produce any crystals. We postulated that by binding into the very long cofactor-binding groove between the two lobes of the acetyltransferase domain, the very long AcCoA molecule can fill this pocket in its entirety and thereby stabilizes the overall protein to an extent that enables crystal growth. In contrast, the highly potent, but much shorter, tool compound WM-8014 (Fig. 1 ▸) cannot stabilize the two lobes of the KAT domain sufficiently for crystallization. To exploit the obtained AcCoA co-crystals of KAT6A, we developed a back-soaking procedure where over the course of nine days, AcCoA co-crystals of KAT6A^mutCys^ were first transferred to reservoir buffer without any ligand to soak out the bound AcCoA and to remove the excess AcCoA present in the original drops. The crystals were then transferred into fresh drops made from reservoir solution supplemented with the respective small-molecule inhibitor first at 5 m*M*, then at 7 m*M* and finally at 10 m*M*. Diffraction data sets of crystals treated this way yielded structures in which AcCoA was systematically still present in chain *A*, while in chain *B* it was successfully replaced by the respective inhibitor (see below). This exchange happened very slowly, over the course of several days. As an example, for WM-8014 a data set collected after only six days of back-soaking showed density in chain *B* which could be fully explained by the new ligand at an occupancy of 0.60 and AcCoA at an occupancy of 0.40 (data set not shown here). Only after ten days of back-soaking (of a different crystal) could the density in chain *A* be fully explained by the new ligand, with no residual difference density suggesting remaining AcCoA in the binding pocket (WM-8014 data set in see Table 1 ▸, density in Fig. 4 ▸).

The asymmetric outcome of the back-soaking experiment – where chain *A* retained AcCoA even after ten days, whereas AcCoA in chain *B* was replaced by the compound of interest – may result from differences in crystal packing around the AcCoA-binding site among the individual protomers. At the inhibitor-binding end of this site, AcCoA itself participates in crystal contacts: in all four protomers the adenine moiety and phosphate group extend towards the solvent and form hydrogen bonds with a neighbouring symmetry-related molecule, involving the side chains of His732′ and Glu738′, as well as the backbone amide NH group of Phe739′. We therefore propose that subtle differences in crystal contacts surrounding the AcCoA-binding site are sufficient to promote AcCoA dissociation in half of the protomers within the asymmetric unit, thereby allowing the compound of interest to enter and bind.

It is important to note that ligand back-soaking into KAT6A^mutCys^ crystals triggered a change in the crystal lattice from four KAT6A protomers per asymmetric unit (chains *A* to *D* in the AcCoA dataset) to two protomers per asymmetric unit (chains *A* and *B* in all back-soaked crystals). Comparison of the two crystal lattices suggests that before back-soaking the four protomers formed two KAT6A homodimers (*A*/*B* and *C*/*D*). During the ten-day back-soaking procedure, the protomers within each homodimer undergo a slight rearrangement, such that the *C*/*D* homodimer becomes a crystal mate of the *A*/*B* homodimer, resulting in a halving of the unit-cell *c* axis. This rearrangement of the crystal packing, together with the associated crystal stress, may be the main reason why the back-soaked crystals diffract to a lower resolution than the original crystals, which were co-crystallized directly with AcCoA. Conversely, the observed lattice change upon ligand soaking indicates that the ligand conformation observed after back-soaking is not an artefact caused by the previously bound AcCoA molecule. Rather, the ligand enforces its own binding mode within the KAT6A binding site, thereby also influencing the crystal lattice.

### 3.2. The crystal structure of KAT6A^mutCys^ in complex with the tool compound WM-8014

We initially tested the new back-soaking procedure reportedhere with the hydrazine-sulfonamide tool compound WM-8014 (Fig. 1 ▸) and were able to solve the co-crystal structure of WM-8014 in complex with KAT6A at a resolution of 2.8 Å (Fig. 4 ▸). WM-8014 binds within the AcCoA-binding pocket in the adenosine and pyrophosphate subpocket. After we determined this crystal structure, Baell and coworkers reported the co-crystal structure of this same KAT6A inhibitor in complex with KAT8^mutant^ (termed MYST^cryst^; Baell *et al.*, 2018 ▸). KAT8^mutant^/MYST^cryst^ is a variant of the catalytic domain of KAT8 in which six active-site residues of KAT8 have been mutated to those found in KAT6A presented in this contribution. We analysed whether this surrogate approach (PDB entry6ba2) had correctly predicted the native binding mode of WM-8014 in KAT6A. The superimposition of both crystal structures in Fig. 4 ▸(*b*) shows that KAT8^mutant^ indeed represents a good surrogate system for KAT6A: the binding mode of WM-8014 is the same in both structures, with the biphenyl moiety inserting deepest into the binding pocket and forming van der Waals contacts with Phe600 and Leu601 (Fig. 4 ▸*a*), and the central hydrazine-sulfonamide group of WM-8014 forming multiple hydrogen-bond interactions with the P-loop of KAT6A (residues 654–659), thereby mimicking the pyrophosphate group of AcCoA. All residues lining the inhibitor-binding site in the KAT6A co-crystal structure adopt the same rotamer conformations as seen in the KAT8^mutant^ surrogate structure.

### 3.3. Exploring the entrance to the inhibitor-binding site: binding modes of quinoline-containing inhibitors in the two crystallization systems

**Figure 5:**
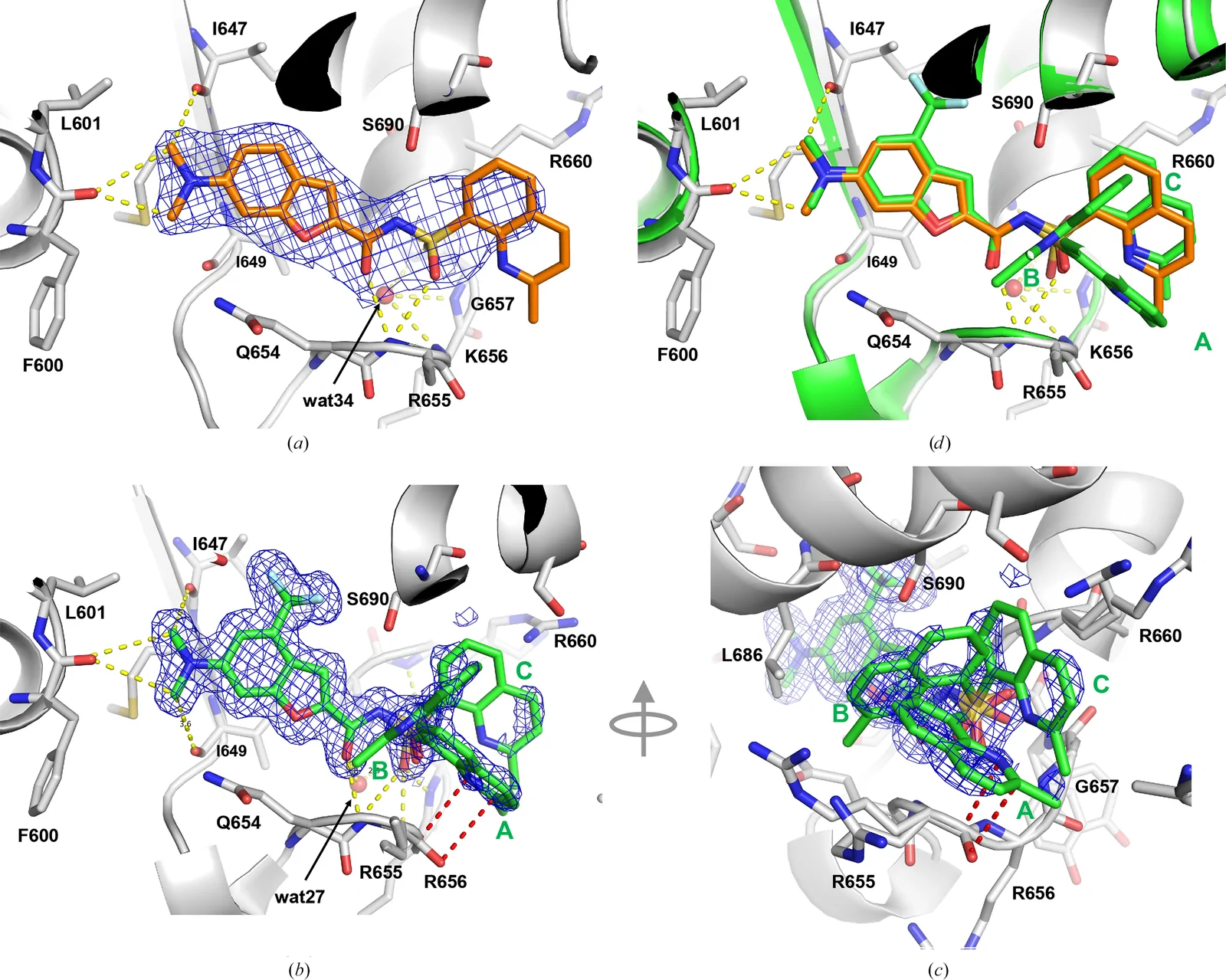
Crystal structures of KAT6A inhibitors with quinoline groups. (*a*) Crystal structure of KAT6A^mutCys^ in complex with compound 1 at 2.7 Å resolution (PDB entry 29us; inhibitor shown with C atoms in orange; 2*F*_o_ − *F*_c_ electron-density map contoured at 1.3σ in blue). (*b*, *c*) Crystal structure of KAT8^mutant^ in complex with compound 2 at 1.4 Å resolution (PDB entry 29ut; inhibitor shown with C atoms in green; 2*F*_o_ − *F*_c_ electron-density map contoured at 1.3σ in blue). The labels A to C in green refer to the three alternative conformations of the quinoline moiety. (*d*) Superimposition of the KAT8^mutant^–compound 2 complex (green) onto the KAT6A^mutCys^–compound 1 complex (orange/grey). Dotted yellow lines indicate hydrogen bonds.

We recently reported the high-throughput screening and optimization campaign which resulted in the discovery of a benzofuran-acylsulfonamide inhibitor series represented by BAY-184 as new inhibitors of KAT6A (Fig. 1 ▸; ter Laak *et al.*, 2024 ▸). During the further optimization of this compound (see also ter Laak *et al.*, 2026 ▸) we tested several eastern extensions to replace the biphenyl moiety in BAY-184. We noticed that quinolines showed high binding potencies, with IC_50_ values of 23 n*M* for compound 1 and 1.5 n*M* for compound 2. To understand the molecular basis for this high potency, we initially back-soaked compound 1 into KAT6A^mutCys^ crystals and obtained a co-crystal structure at 2.7 Å resolution (Tables 1 ▸ and 2 ▸). The structure (Figs. 5 ▸*a* and 5 ▸*b*) reveals a conserved binding mode for the benzofuran scaffold and for the central acylsulfonamide group compared with the biphenyl variant BAY-184 (PDB entry 9fkr). In brief, the benzofuran scaffold inserts into a hydrophobic pocket and is sandwiched between the side chains of residues Ile649 and Leu686. The dimethylamino moiety fills a small subpocket in front of Leu601 and the backbone carbonyl O atom of Phe600, and its two methyl groups donate nonclassical C—H⋯O hydrogen bonds to the backbone carbonyl O atoms of Phe600 (3.5 and 3.6 A), of Ile647 (3.6 A) and of Ile649 (3.6 A). Importantly, the acylsulfonamide group mimics the pyrophosphate unit of AcCoA and accepts multiple hydrogen bonds from backbone amide N atoms of the P-loop (residues 655–660).

Despite the presence of well defined electron density for most of the molecule, only very weak density was observed for the quinoline moiety, which was therefore modelled tentatively. We interpreted this weak density as an indication that the quinoline group adopts two or more alternative conformations that cannot be resolved at the moderate resolution of 2.7 Å. Support for this interpretation was obtained when the related quinoline compound 2 (Figs. 5 ▸*b*–5 ▸*d*) was co-crystallized with KAT8^mutant^/MYST^cryst^, yielding a dataset at 1.4 Å resolution (Fig. 5 ▸*b*). Superposition of the structures (Fig. 5 ▸*d*) showed that the two quinoline compounds share the same binding mode with respect to the benzofuran scaffold and the acylsulfonamide group, further confirming that KAT8^mutant^/MYST^cryst^ can serve as a surrogate for KAT6A.

At 1.4 Å resolution, the electron-density maps clearly revealed that the quinoline group adopts three alternative conformations, labelled A, B and C in Figs. 5 ▸(*b*)–5 ▸(*d*). These conformations were modelled with occupancies of 0.40, 0.40 and 0.20, respectively. Conformation C corresponds to the conformation tentatively modelled in the medium-resolution KAT6A structure with compound 1. In addition, conformations A and C of the quinoline ring in the KAT8^mutant^/MYST^cryst^ structure are identical to the two alternative conformations adopted by the first phenyl ring of the biphenyl compound BAY-184 (Fig. 1 ▸) when bound to KAT6A^mutCys^ (ter Laak *et al.*, 2024 ▸). At this end of the inhibitor-binding site, the cavity opens towards the solvent, and the residues lining its wall provide alternative contact areas for an aromatic moiety. In the structure of quinoline compound 2, conformations A and C form alternative stacking interactions with the side chains of Arg655 and Arg660, respectively. Both stacking interactions are also observed in the KAT6A–BAY-184 structure, in which the inner phenyl ring of the biphenyl group occupies the position of the benzo portion of the quinoline moiety (PDB entry 9fkr; not shown in Fig. 5 ▸).

Interestingly, in addition to conformations A and C, the quinoline group of compound 2 can adopt a third conformation, B, with an occupancy comparable to that of conformation A. In this conformation, the quinoline moiety and its methyl substituent extend into a hydrophobic subpocket formed by the side chains of Leu686 and Arg655. Judging from the occupancy, this interaction appears to provide a similarly favourable contribution to binding as the stacking interaction formed by conformation A with the side chain of Arg655 (Fig. 5 ▸).

In summary, the substantial difference in resolution between the KAT8^mutant^/MYST^cryst^ and KAT6A^mutCys^ structures (1.4 Å versus 2.7 Å, respectively) must be taken into account. The lower resolution of the KAT6A^mutCys^ dataset may obscure additional factors, such as radiation damage, that could contribute to the lack of density for the quinoline group. Nevertheless, the conformational flexibility observed in the KAT8^mutant^/MYST^cryst^ structure provides a plausible explanation for the weak or absent quinoline density in the lower resolution KAT6A^mutCys^ dataset.

The higher occupancy of conformation A over C for compound 2 may in part be based on an additional dipole–dipole interaction observed only for conformation A (red dotted lines in Figs. 5 ▸*b* and 5 ▸*c*). The quinoline ring N atom and its adjacent C atom (carrying the methyl side chain) form a dipole which binds parallel to an opposite dipole formed by the backbone carbonyl group of Arg655. We have recently observed a very similar dipole–dipole interaction in the same position for the further optimized compound BAY-7728 co-crystallized with KAT8^mutant^/MYST^cryst^ (ter Laak *et al.*, 2026 ▸): In BAY-7728, the quinoline group of compound 2 is replaced with an *ortho*-ethoxyphenyl group. The –O-CH_2_– section of the ethoxy group acts as a dipole and stacks in the same way against the carbonyl group as does the –N-CH– section of the quinoline ring system in compound 2.

The co-crystal structure of compound 2 shows that the trifluoromethyl group in position 4 of the benzofuran ring fills a hydrophobic subpocket lined by Ile647, Gly687 and the hydrophobic section of the side chain of Ser690 (Fig. 5 ▸*b*). A superimposition of the structures of KAT6A^mutCys^ soaked with compound 1 and of KAT8^mutant^ co-crystallized with compound 2 (Fig. 5 ▸*d*) shows that the CF_3_ group fills the subpocket without changing the overall conformation of the ligand or of the binding pocket. Notably, compound 1 and compound 2 differ only in the presence or absence, respectively, of this CF_3_ group. Filling this subpocket must therefore be responsible for the 15-fold increase in potency in the biochemical assay from IC_50_ = 23 n*M* for compound 1 to IC_50_ = 1.5 n*M* for compound 2 (Fig. 1 ▸). In the recently published co-crystal structure of the closely related inhibitor BAY-7728 (ter Laak *et al.*, 2026 ▸), this same subpocket was filled with an F atom, which also triggered a significant improvement in potency.

### 3.4. Crystal structure of CTX Example 41 in complex with KAT8^mutant^

**Figure 6:**
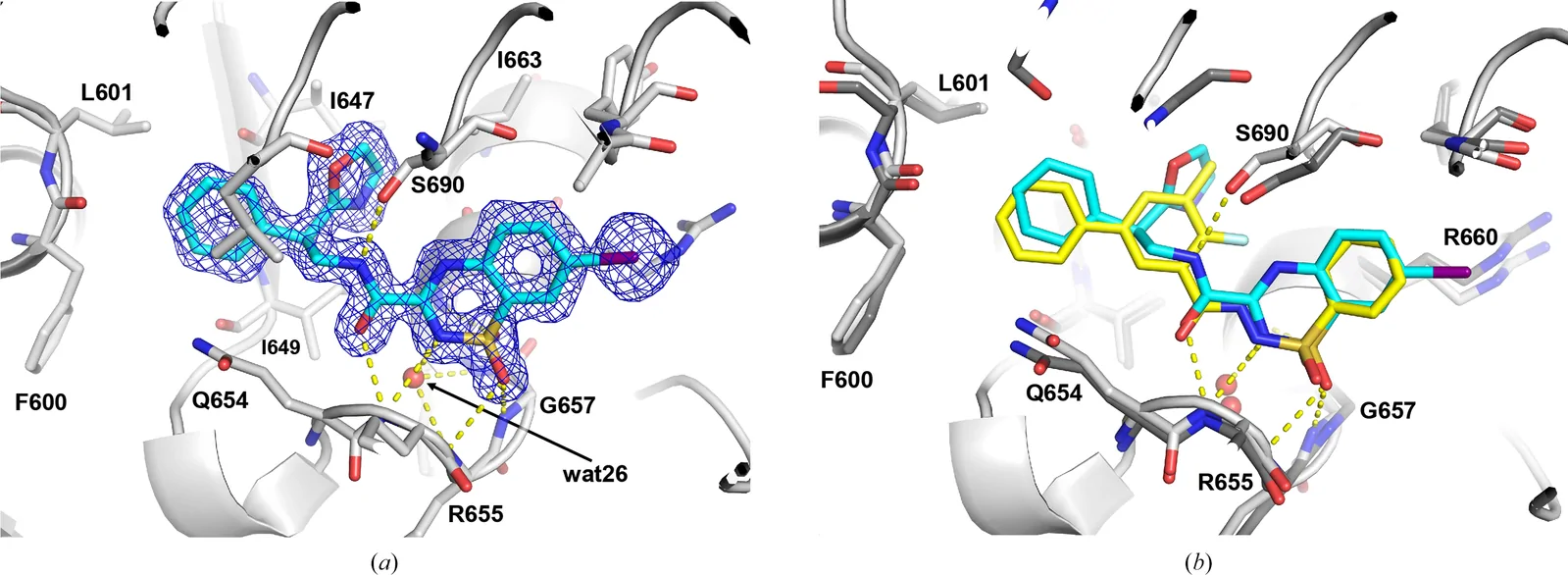
Crystal structure of KAT8^mutant^/MYST^cryst^ in complex with Example 41. (*a*) The inhibitor Example 41 (Morrow *et al.*, 2018 ▸; later termed compound 29n; Suwandi *et al.*, 2026 ▸) is depicted in stick representation with C atoms in cyan (PDB entry 29uu). The final 2*F*_o_ − *F*_c_ electron-density map (1.3 Å resolution, contoured at 1.3σ) is shown in blue. Hydrogen bonds are depicted as yellow dotted lines. (*b*) Superimposition of the previously published crystal structure of KAT8^mutant^/MYST^cryst^ in complex with inhibitor WM-8014 (PDB entry 6ba2; Baell *et al.*, 2018 ▸), depicted in stick representation with C atoms in yellow.

During the optimization of the benzofuran-acylsulfonamide inhibitor series represented by BAY-184, compound 1 and compound 2 (Fig. 1 ▸), Morrow and coworkers reported benzo-[1,2,4]thiadiazine compounds as a further class of small-molecule KAT6A inhibitors different from our benzofuran inhibitor series and from the CTX hydrazone-sulfonamides (represented by WM-8014) (Fig. 1 ▸). In order to understand the binding mode of this series, we synthesized the benzo-[1,2,4]thiadiazine Example 41 (Fig. 1 ▸), which was first reported in a patent application (Morrow *et al.*, 2018 ▸) as a very potent KAT6A inhibitor (IC_50_ = 5 n*M*). Recently, Suwandi and coworkers presented a structure–activity relationship study of this compound class and reported Example 41 as Compound 27n (Suwandi *et al.*, 2026 ▸), with an IC_50_ value of 5 n*M* for the racemate, IC_50_ = 2 n*M* for the eutomer and IC_50_ = 29 n*M* for the distomer (Suwandi *et al.*, 2026 ▸). We synthesized Example 41 as described by Morrow and coworkers, separated the enantiomers and determined the co-crystal structure of the eutomer in complex with KAT8^mutant^. The crystal structure, solved at 1.3 Å resolution (Fig. 6 ▸), reveals that it binds in the same binding pocket as observed previously for WM-8014 and BAY-184 at the ‘adenosyl end’ of the long AcCoA-binding pocket. The density map revealed unambiguously that the co-crystallized compound features the (*R*) configuration. Interestingly, the recently published co-crystal structure (PDB entry 9ooj) of KAT8^mutant^ in complex with the closely related compound 18c from Suwandi *et al.* (2026 ▸) features the (*S*) configuration, which means that the oxazole and the phenyl ring at the stereo centre have swapped positions. At the very high resolution of our structure, the density maps unambiguously confirmed that the six-membered phenyl ring and the five-membered oxazole ring have been modelled correctly. It may be, however, that for Example 41 co-crystallized by us the eutomer is represented by the (*R*) enantiomer, while for compound 27n co-crystallized by Suwandi and coworkers the (*S*) enantiomer is the more potent variant.

Superimposition of the crystal structures of Example 41 and WM-8014 (Fig. 6 ▸*b*) reveals strong structural similarity in the binding mode. Indeed, Example 41 is related to WM-8014 and may be the outcome of an intensive redesign. The outer phenyl ring of both compounds inserts deepest into the inhibitor-binding pocket of KAT6A and occupies the same subpocket, sandwiched between Met648 in the back and Leu686 (both residues are not shown in Fig. 6 ▸ for clarity). The adjacent methylfluorophenyl ring of WM-8014 has been opened and replaced by the oxazole side chain of Example 41, which fills the same subpocket as one of the two methyl groups of WM-8014, lined by Ile647, Ile649 and Ile663. The central hydrazone-sulfonamide unit of WM-8014 has been replaced by a flipped amide, and a ring closure introduced into the outer phenyl-sulfonamide has generated the new thiaquinazoline-4,4-dione heterocycle. The sulfonamide unit is now integrated as a sulfam in the thiaquinazoline-4,4-dione ring system, but maintains its exact position in the binding pocket and binds to the P-loop of KAT6A (residues 654–660) in the same way as the sulfonamide group of WM-8014. It forms the same series of hydrogen bonds to the backbone amide N atoms of Arg655, Gly657, Tyr658, Gly659 and Arg660, which also include a conserved water molecule (wat26), and thereby takes over the important role of mimicking the two phosphate groups of the AcCoA cofactor.

### 3.5. Exploring the KAT6A inhibitor-binding site via crystal structures from three different chemical series

**Figure 7:**
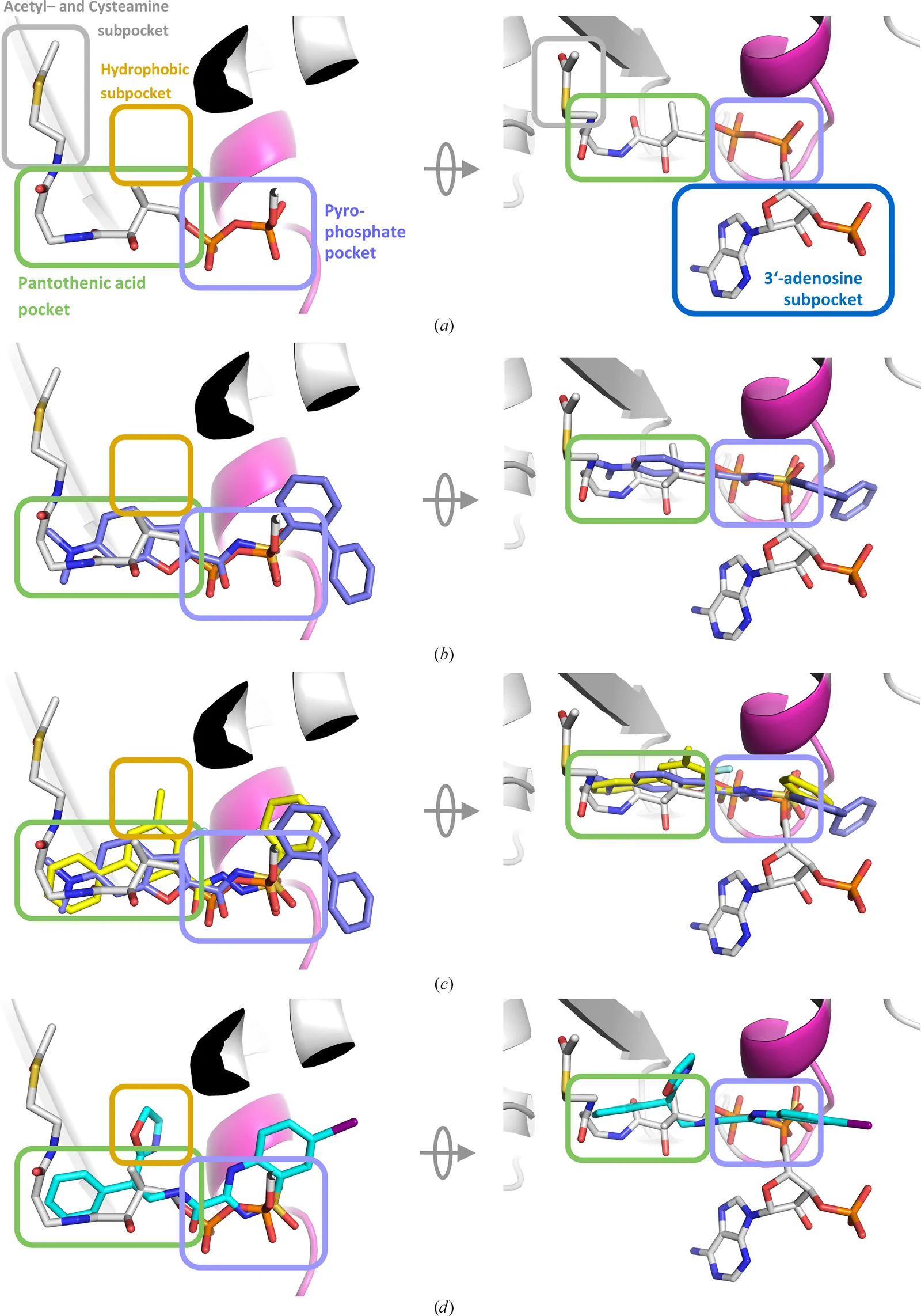
Comparative binding-mode analysis over three different inhibitor classes. (*a*) shows KAT6A^mutCys^ in complex with the cofactor AcCoA (PDB entry 29uq, C atoms in grey). The P-loop and the beginning of the adjacent helix which coordinate the pyrophosphate unit of the cofactor are shown in magenta. (*b*) shows the superimposition of KAT6A^mutCys^–AcCoA (PDB entry 29ur; C atoms in grey) with KAT6A^mutCys^ in complex with benzofuran BAY-184 (PDB entry 9fkr; ter Laak *et al.*, 2024 ▸; C atoms in lilac); (*c*) shows the same superimposition as in (*b*), but now additionally with the co-complex of KAT6A^mutCys^ with WM-8014 of the hydrazine-sulfonamide series (PDB entry 29uq; C atoms in yellow). (*d*) shows the superimposition of KAT6A^mutCys^–AcCoA with KAT8^mutant^/MYST^cryst^ in complex with the benzothiadiazine Example 41 (PDB entry 29uu; C atoms in cyan).

Crystal structures of KAT6A in complex with its cofactor AcCoA (Holbert *et al.*, 2007 ▸, PDB entry 2ozu and this work) revealed that the elongated cofactor inserts into a long, deep and partly solvent-excluded surface groove between the two lobes of the enzyme (Figs. 3 ▸*a* and 7 ▸*a*). In this work, we report co-crystal structures of three chemical small-molecule inhibitor classes, the benzofuran-acylsulfonamide series [represented by BAY-184 (ter Laak *et al.*, 2024 ▸), BAY-7728 (ter Laak *et al.*, 2026 ▸) and compounds 1 and 2 in this work], the acylsulfonylhydrazide series [represented by WM-8014 (Baell *et al.*, 2018 ▸) and this work] and the 1,2,4-thiadiazine series [represented by Example 41/compound 27n (Morrow *et al.*, 2018 ▸; Suwandi *et al.*, 2026 ▸) and this work]. A series of superimpositions of the inhibitor-bound structures onto AcCoA-bound KAT6A^mutCys^ revealed which parts of the AcCoA pocket are targeted by the individual inhibitors (Fig. 7 ▸). The AcCoA cofactor (Fig. 7 ▸*a*) can be subdivided into (i) a deeply buried acetyl-cysteamine group which inserts deepest into the groove, (ii) a pantothenic acid moiety which occupies the middle section of the elongated binding pocket, (iii) the negatively charged pyrophosphate group which is tightly coordinated via multiple hydrogen bonds to the P-loop (residues 655–660, magenta in Fig. 7 ▸*a*) and (iv) an outer 3′-phosphoadenosine group which protrudes from the binding groove towards the solvent and is relatively loosely coordinated, as seen by two different binding conformations in PDB entries 2rc4 (Holbert *et al.*, 2007 ▸) and 2ozu.

The superimpositions in Figs. 7 ▸(*b*)–7 ▸(*d*) reveal that all three inhibitor classes use their sulfonamide-containing central sections to bind to the P-loop and to thereby mimic the pyrophosphate group of AcCoA, forming multiple hydrogen bonds to the P-loop. The mostly hydrophobic pantothenic acid pocket is filled with the benzofuran scaffold and its dimethylamino decoration in the case of our series (BAY-184 in Figs. 7 ▸*b* and 7 ▸*c*), the biphenyl section of WM-8014 of the acylsulfonylhydrazide series (Fig. 7 ▸*c*) and the phenylethyl group of Example-41 of the thiadiazine series (Fig. 7 ▸*d*).

**Figure 8:**
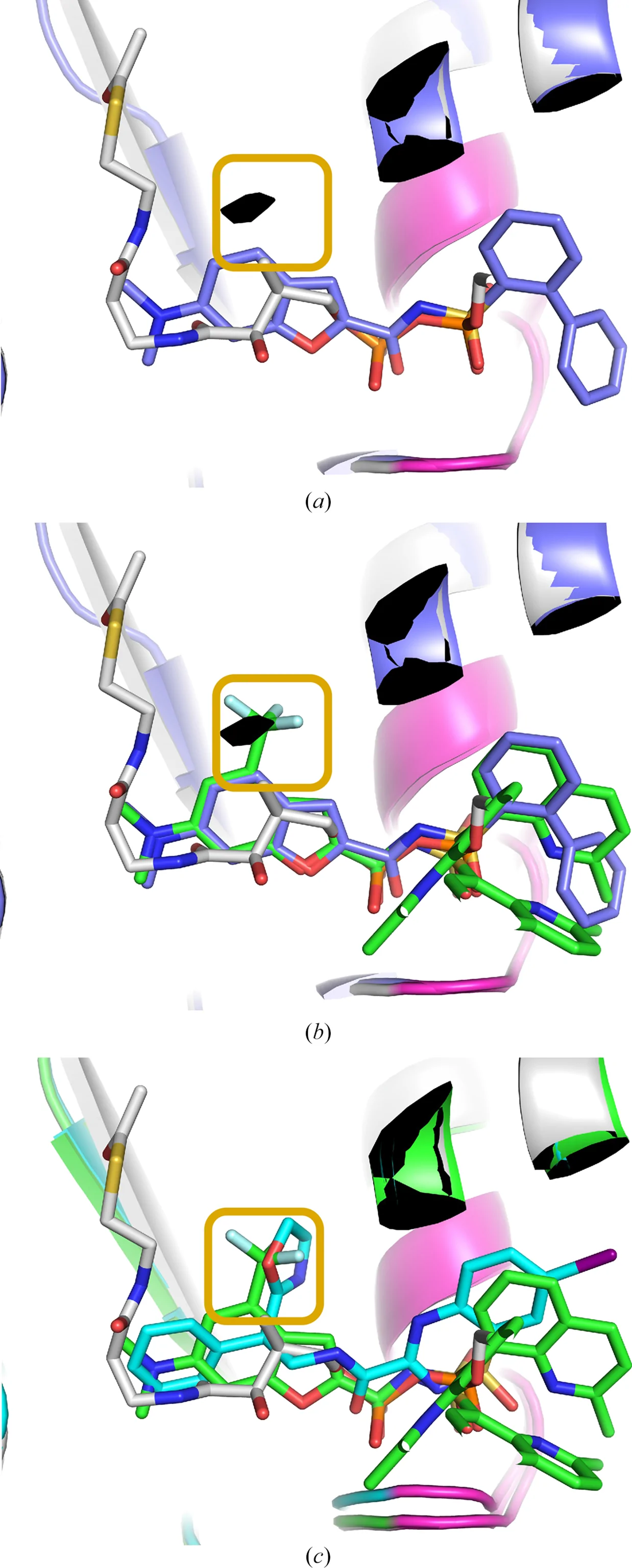
Exploiting the hydrophobic side pocket in the benzofuran-acylsulfonamide series and in the benzothiadiazine series. (*a*) shows the superimposition of KAT6A^mutCys^ in complex with AcCoA (PDB entry 29uq; C atoms in grey) and in complex with BAY-184 (PDB entry 9fkr; ter Laak *et al.*, 2024 ▸; C arbon atoms in lilac). The location of a hydrophobic side pocket is indicated by an orange box. (*b*) shows the same superimposition as in (*a*), but now additionally with the co-complex of KAT8^mutant^/MYST^cryst^ and compound 2 (PDB entry 29ut; C atoms in green); (*c*) shows the superimposition of KAT6A^mutCys^ in complex with AcCoA and the co-complex structures of KAT8^mutant^/MYST^cryst^ with compound 2 (PDB entry 29ut; C atoms in green) and with the benzo-thiadiazine Example 41 (PDB entry 29uu; C atoms in cyan).

Interestingly, none of the three inhibitor series reaches into the deepest part of the pocket: the acetyl and cysteamine subpocket (Fig. 7 ▸*a*). We explored this approach in our benzofuran-acylsulfonamide series, but were not successful (for details, see ter Laak *et al.*, 2026 ▸). However, the more optimized variants WM-8014 and Example-41 reach out and fill the hydrophobic subpocket (orange box in Fig. 7 ▸) which is not targeted by AcCoA. This is depicted in more detail in Fig. 8 ▸, which shows that our early compound BAY-184 was still lacking a group exploiting the hydrophobic subpocket (Fig. 8 ▸*a*), while the further optimized compound 2 (Fig. 8 ▸*b*) and also the even further optimized inhibitor BAY-7728 (ter Laak *et al.*, 2026 ▸) have been grown to fill this pocket. Also, Example 41 occupies this pocket, here with an imidazole ring (Fig. 8 ▸*c*).

The funnel-like wider opening at the adenosyl end of the AcCoA-binding groove allows different ways to occupy this wider space, which is consistent with the observed two binding conformations of the adenosine group in AcCoA structures (PDB entries 2ozu and 2rc4; not shown here). This is also reflected by several of the small-molecule inhibitors which show alternative conformations for their respective outer groups: the biphenyl group in BAY-184 with two alternative conformations in chains *A* and *B* (PDB entry 9fkr; Ter Laak *et al.*, 2024 ▸) and the three alternative conformations of the outer quinoline group of compound 2 (PDB entry 29ut; Figs. 5 ▸*b*–5 ▸*d*).

## 4. Summary and outlook

Wild-type KAT6A proved to be a challenging crystallization target. Whereas early structures were reported only in complex with AcCoA, and not for the apo protein or inhibitor-bound states (Holbert *et al.*, 2007 ▸; PDB entry 2ozu), we were unable to reproduce these crystals. Crystallization was only achieved after engineering a surface mutant, KAT6A^mutCys^, in which four solvent-exposed cysteine residues were replaced by serine residues. KAT6A^mutCys^ crystallized readily with AcCoA, and subsequent back-soaking experiments to remove AcCoA allowed the determination of several inhibitor-bound structures. This allowed us to the determine the first reported KAT6A co-crystal structure with WM-8014, which confirmed the validity of the previously published KAT8^mutant^/MYST^cryst^ surrogate co-crystal structure with WM-8014 (Baell *et al.*, 2018 ▸).

KAT6A^mutCys^ was further used to obtain co-crystal structures with BAY-184 (ter Laak *et al.*, 2024 ▸) and compound 1 (this study), but the moderate resolution of this crystal system, particularly after back-soaking (2.7–2.95 Å), limited detailed interpretation. By contrast, KAT8^mutant^/MYST^cryst^ crystallized more readily, allowed direct co-crystallization and consistently yielded substantially higher resolution structures (1.3–1.8 Å), in agreement with other reports (Baell *et al.*, 2018 ▸; Suwandi *et al.*, 2026 ▸). The high-resolution structures were critical for resolving conformational flexibility in the right-hand side of the benzofuran inhibitor series, which was not defined for compound 1 in the 2.6 Å resolution KAT6A structure but was clearly visible for the closely related compound 2 in the 1.4 Å resolution KAT8^mutant^/MYST^cryst^ structure.

Overall, both the new approach to crystallize KAT6A as the surface-cysteine mutant KAT6A^mutCys^ and the new binding modes determined for the inhibitors co-crystallized in the structures reported here will help to accelerate further inhibitor design for KAT6A as an anticancer drug target.

## Supplementary Material

PDB reference: KAT6A^mutCys^–AcCoA, 29uq

PDB reference: KAT6A^mutCys^–WM-8014, 29ur

PDB reference: KAT6A^mutCys^–compound 1, 29us

PDB reference: KAT8^mutant^/MYST^cryst^–compound 2, 29ut

PDB reference: KAT8^mutant^/MYST^cryst^–Example 41, 29uu

## Figures and Tables

**Table 1:** KAT6A^mutCys^ and KAT8^mutant^ data-collection and processing statistics Values in parentheses are for the outer shell.

Data set	KAT6A^mutCys^–AcCoA	KAT6A^mutCys^–WM-8014	KAT6A^mutCys^–compound 1	KAT8^mutant^–compound 2	KAT8^mutant^–Example 41
PDB code	29uq	29ur	29us	29ut	29uu
Diffraction source	BL14.1, BESSY	BL14.1, BESSY	P11, PETRA	P11, PETRA	P11, PETRA
Wavelength (Å)	0.9184	0.9184	1.0332	1.0332	1.0332
Temperature (K)	100	100	100	100	100
Detector	PILATUS	PILATUS	PILATUS	PILATUS	PILATUS
Crystal-to-detector distance (mm)	293	495	275	154.6	154.6
Rotation range per image (°)	0.1	0.1	0.1	0.1	0.1
Total rotation range (°)	200	360	180	180	120
Space group	*P*1	*P*1	*P*1	*P*2_1_2_1_2_1_	*P*2_1_2_1_2_1_
*a*, *b*, *c* (Å)	37.3, 64.6, 115.6	36.2, 57.6, 62.5	37.0, 58.9, 64.9	46.3, 57.4, 121.1	46.5, 57.7, 121.2
α, β, γ (°)	85.6, 85.2, 89.6	94.1, 90.3, 103.2	93.0, 91.3, 102.9	90.0, 90.0, 90.0	90.0, 90.0, 90.0
Mosaicity (°)	0.33	0.61	0.23	0.068	0.047
Resolution range (Å)	44.59–2.13 (2.26–2.13)	43.27–2.78 (2.95–2.78)	44.25–2.70 (2.86–2.70)	46.27–1.40 (1.48–1.40)	46.46–1.30 (1.38–1.30)
Total No. of reflections	112902	40973	24922	420835	347300
No. of unique reflections	54078	11480	13855	64350	79136
Completeness (%)	90.7 (89.2)	93.5 (90.0)	94.1 (94.6)	99.9 (99.8)	97.7 (96.1)
Multiplicity	2.1 (2.1)	3.6 (3.4)	1.7 (1.7)	6.5 (6.5)	4.4 (4.2)
〈*I*/σ(*I*)〉	7.3 (1.8)	7.5 (0.99)	6.7 (1.4)	15.7 (1.3)	11.5 (1.3)
*R*_merge_	0.082 (0.463)	0.133 (1.093)	0.137 (0.598)	0.057 (1.401)	0.056 (0.827)
*R*_r.i.m._	0.110 (0.626)	0.156 (1.283)	0.194 (0.846)	0.062 (1.355)	0.064 (0.943)
CC_1/2_ (%)	99.5 (74.4)	99.3 (65.1)	97.5 (58.0)	99.9 (63.7)	99.8 (60.5)
Overall *B* factor from Wilson plot (Å^2^)	35.6	59.9	49.9	25.5	22.5

**Table 2:** Structure solution and refinement Values in parentheses are for the outer shell.

Data set	KAT6A^mutCys^–AcCoA	KAT6A^mutCys^–WM-8014	KAT6A^mutCys^–compound 1	KAT8^mutant^–compound 2	KAT8^mutant^–Example 41
PDB code	29uq	29ur	29us	29ut	29uu
Resolution range (Å)	44.59–2.13 (2.19–2.13)	43.27–2.78 (2.86–2.78)	34.19–2.70 (2.77–2.70)	41.65–1.40 (1.44–1.40)	46.46–1.30 (1.38–1.30)
Completeness (%)	90.72	93.5	91.4	99.9	97.7
σ Cutoff	n/a	n/a	n/a	n/a	n/a
No. of reflections, working set	51976 (3712)	10902 (629)	12969 (979)	62249 (4532)	77035 (5490)
No. of reflections, test set	2100 (150)	574 (36)	432 (32)	2100 (153)	2101 (150)
Final *R*_work_	0.225 (0.309)	0.278 (0.434)	0.235 (0.317)	0.129 (0.321)	0.124 (0.296)
Final *R*_free_	0.275 (0.318)	0.329 (0.460)	0.309 (0.359)	0.178 (0.314)	0.161 (0.347)
Cruickshank DPI	0.451	0.541	0.408	0.049	0.038
No. of non-H atoms					
Protein, chain *A*/*B*/*C*/*D*	2290/2267/2264/2260	2222/2227/—/—	2265/2263/—/—	2385/—/—/—	2366/—/—/—
AcCoA, chain *A*/*B*/*C*/*D*	51/51/51/51	51/—/—/—	—	—	—
Zinc ion, chain *A*/*B*/*C*/*D*	1/1/1/1	1/1/—/—	1/—/—/—	1/—/—/—	1/—/—/—
Inhibitor, chain *A*/*B*	—/—	—/27	29/29	61/—	29/—
Chloride ions	—	—	—	2	5
Glycerol	18	—	—	12	24
PEG molecule	7	—	—	—	7
Water	630	46	37	270	333
Total	9986	4675	4625	2734	2765
R.m.s. deviations					
Bond lengths (Å)	0.006	0.002	0.017	0.018	0.016
Angles (°)	1.359	1.149	1.869	1.781	1.832
Average *B* factors (Å^2^)					
Overall	37.4	70.4	68.1	31.1	25.9
Protein, chain *A*/*B*/*C*/*D*	33.4/49.0/43.1/34.3	79.1/71.4/—/—	65.5/71.9/—/—	30.6/—/—/—	23.8/—/—/—
Zn ion, chain *A*/*B*/*C*/*D*	23.4/22.8/21.8/22.3	40.0/37.1/—/—	47.0/49.8/—/—	15.8/—/—/—	13.8/—/—/—
AcCoA, chain *A*/*B*/*C*/*D*	27.7/35.7/31.6/26.0	80.9/—/—/—	—	—	—
Inhibitor, chain *A*/*B*	—/—	—/59.0	37.8/41.9	20.1/—	16.2/—
Chloride ion	—	—	—	25.2	24.7
Glycerol	44.7	—	—	35.3	24.3
PEG molecule	55.7	—	—	—	54.2
Water	37.1	40.0	45.7	41.6	40.3
Ramachandran plot					
Most favoured (%)	95.9	89.4	90.8	96.8	96.4
Allowed (%)	99.3	99.0	97.9	99.6	99.6
